# Efficacy and safety of tenofovir alafenamide in HBeAg-positive and negative chronic hepatitis B patients aged over 30 years with normal ALT and detectable HBV DNA: a 96-week retrospective two-center study

**DOI:** 10.3389/fmed.2026.1764956

**Published:** 2026-07-23

**Authors:** Yanfang Li, Jing Sun, Xinxin Liu, Haifeng Yu, Youde Liu, Ning Sun

**Affiliations:** 1 Department of Hepatology, Yantai Qishan Hospital, Yantai, China; 2 Department of Gastroenterology, Yantai Yuhuangding Hospital, Yantai, China

**Keywords:** chronic hepatitis B, HBV DNA, renal safety, retrospective study, tenofovir alafenamide, virological response

## Abstract

**Background:**

The 2022 update of Chinese Guidelines for Chronic Hepatitis B (CHB) Management advocates initiating antiviral therapy regardless of alanine aminotransferase (ALT) status in HBV DNA-positive patients aged > 30 years. This study aimed to investigate the virological response and renal safety profile of tenofovir alafenamide (TAF) in this specific population through both short-term (48-week) and extended (96-week) observations.

**Methods:**

In this multicenter retrospective cohort study, 122 treatment-naïve CHB patients meeting inclusion criteria from two tertiary hospitals (Yantai Qishan Hospital and Yantai Yuhuangding Hospital) underwent standard TAF monotherapy (25 mg/day). Serial assessments of virological parameters (HBV DNA quantification) and renal function markers (serum creatinine, estimated glomerular filtration rate) were conducted at baseline, week 48, and week 96.

**Results:**

After 48 weeks of TAF treatment, 74.6% of the 122 enrolled patients achieved HBV DNA conversion (HBV DNA < 20 IU/mL), with the difference in conversion rates being statistically significant (*P* < 0.001). Among the 80 patients who completed 96 weeks of follow-up, a progressive increase in HBV DNA conversion rates was observed between week 48 and week 96, demonstrating statistically significant improvement over time (*P* < 0.001). Intergroup comparisons showed no statistically significant differences, and all safety parameters exhibited no clinically relevant changes during the trial.

**Conclusion:**

This real-world evidence demonstrates durable antiviral efficacy of TAF in guideline-defined CHB patients aged > 30 years with normal ALT and active viremia, accompanied by favorable renal tolerability throughout the 96-week treatment period. Extended follow-up is ongoing to establish long-term clinical outcomes.

## Introduction

Chronic hepatitis B (CHB) continues to pose a significant global health challenge, with an estimated 296 million individuals affected worldwide and approximately 1.5 million new infections annually (1). Long-term infection substantially elevates the risk of developing cirrhosis and hepatocellular carcinoma (HCC). In China—a region historically burdened with high HBV prevalence—national epidemiological surveillance has revealed a progressive decline in infection rates over the past three decades. Currently, the prevalence rate among adults is approximately 3% (2). Despite these improvements, intensified therapeutic strategies remain imperative to mitigate the risks of advanced liver disease and mortality.

Current international guidelines exhibit discordance regarding patient selection criteria for antiviral therapy. While both the 2015 Asia-Pacific Association for the Study of the Liver guidelines (3) and the 2018 American Association for the Study of Liver Diseases (AASLD) recommendations (4) advise against initiating antiviral treatment in patients with normal alanine aminotransferase (ALT) levels, emerging evidence challenges this paradigm. Compelling data demonstrate that individuals with normal ALT yet active viral replication frequently exhibit significant hepatic fibrosis progression (5) and face heightened risks of HCC development and all-cause mortality (6, 7). As our understanding of CHB continues to deepen, the treatment decisions for patients with normal ALT levels often depend on age, viral load, degree of liver fibrosis, family history, and other risk factors. This evolving understanding has prompted guideline revisions to incorporate case-specific antiviral initiation, particularly for populations excluded from prior recommendations but requiring urgent intervention to improve long-term outcomes—a critical step toward achieving the WHO’s 2030 target for eliminating viral hepatitis as a public health threat.

Clinically, patients in the “indeterminate phase” of chronic HBV infection (distinct from classical natural history stages) demonstrate accelerated disease progression, necessitating therapeutic intervention (8, 9). Reflecting these insights, China’s 2022 updated HBV management guidelines (1) introduced substantial revisions: (1) refined stratification of HBV natural history phases; (2) expanded treatment eligibility encompassing both HBeAg-positive and -negative patients; and (3) incorporation of age > 30 years as an independent treatment criterion for individuals with detectable serum HBV DNA and normal ALT levels—a departure from the 2019 guidelines that restricted eligibility to patients with hepatic fibrosis progression, cirrhosis, or familial HCC history. This paradigm shift aligns with epidemiological findings from mainland China, where individuals aged > 30 years exhibit markedly elevated liver-related mortality risks (10), underscoring age as a pivotal determinant in therapeutic decision-making. Furthermore, tenofovir alafenamide (TAF) has been endorsed as a first-line antiviral agent across all eligible populations, supported by robust safety profiles in clinical practice. Previous real-world studies have shown that TAF can be used to treat CHB patients with normal ALT levels, with lower renal safety concerns and a reduced incidence of adverse events (11). Therefore, this study focuses specifically on the use of TAF in the patient population under investigation.

Despite these guideline updates, real-world evidence validating the therapeutic efficacy of expanded treatment criteria—particularly in the > 30-year-old population with normal ALT—remains scarce. Thus, the main objective of this study is to investigate the efficacy of TAF in individuals over 30 years old with normal ALT levels, and to also examine its impact on renal function. These findings are anticipated to provide critical evidence supporting China’s revised guidelines and inform global HBV management strategies.

## Materials and methods

### Patient selection

**FIGURE 1:**
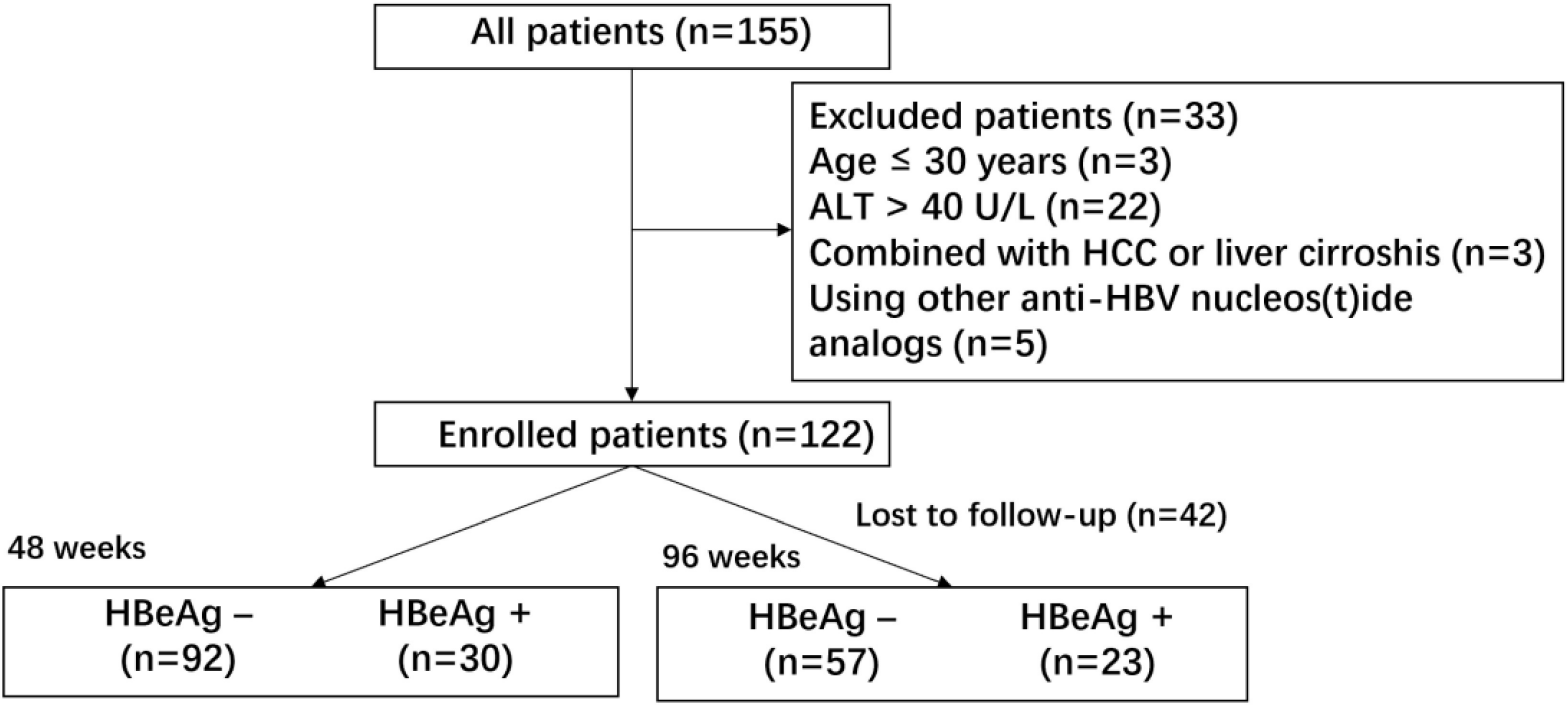
The screening flow chart of enrolled patients.

A total of 122 patients with chronic hepatitis B (CHB) were consecutively enrolled from Yantai Qishan Hospital and Yantai Yuhuangding Hospital. The inclusion criteria were: (1) Age > 30 years; (2) Detectable serum HBV DNA ( ≥ 20 IU/mL); (3) At least three alanine aminotransferase (ALT) measurements below the upper limit of normal (ULN, 40 U/L) within 1 year, with intervals ≥ 3 months (12). (4) Treatment-naïve to other anti-HBV nucleos(t)ide analogs or interferon therapy; (5) Minimum follow-up duration of 48 weeks. Exclusion criteria included: (1) Confirmed liver cirrhosis, hepatocellular carcinoma (HCC), or other liver diseases (e.g., autoimmune hepatitis) based on the results of imaging examinations and clinical diagnoses; (2) Family history of cirrhosis or HCC; (3) Coinfection with human immunodeficiency virus (HIV) or hepatitis C virus (HCV); (4) Pregnancy or comorbid psychiatric disorders. The final list of included patients is shown in the following Figure 1. A total of 122 patients were enrolled. After 48 weeks of follow-up, all 122 patients were still in the study; after 96 weeks of follow-up, 80 patients remained.

### Sample size calculation

Previous studies showed that the CVR rate to TAF treatment in CHB patients ranged from 60% to 80%. This study adopted a median value of 70% for sample size estimation based on α = 0.05, β = 0.2, and a dropout rate of 20%, yielding a required sample size of 114. Ultimately, 122 patients were followed up in this study, meeting the statistical requirements.

### Study design and grouping

This retrospective cohort study used the pre-TAF clinical status of each patient as the self-control baseline. A total of 122 patients were included, all patients took their medication regularly during the follow-up period, and their virological response as well as hepatic and renal function were evaluated at multiple time points during TAF treatment. Patients were further stratified into two subgroups according to baseline HBeAg status: HBeAg-positive (*n* = 30) and HBeAg-negative (*n* = 92). The effect of baseline HBeAg status on antiviral efficacy was subsequently compared between the two groups. Demographic characteristics, including sex, age, as well as virological markers (HBeAg, HBsAg, and HBV DNA) and safety-related parameters (platelet count, lipid profile, AST, ALT, serum creatinine, eGFR, and CKD stage), were collected at baseline and at weeks 48 and 96 after initiation of TAF therapy.

### Laboratory assays

HBV DNA quantification: Performed using a fully automated nucleic acid purification and fluorescence PCR system (Roche Diagnostics; lower detection limit: 20 IU/mL). Biochemical tests: ALT, AST, and other markers were analyzed with an AU5800 automated analyzer (Zhejiang Yikang Biotech reagents; ALT/AST detection limits: 40 U/L and 35 U/L, respectively). Bilirubin: Measured using Beckman Coulter Laboratory Systems (Suzhou) kits.

### Outcome measures

Primary endpoint: Virologic response, also known as HBV DNA conversion, it refers to the situation where the level of HBV DNA is below the detection limit (Abbott Molecular Inc., 20 IU/mL) at 48 and 96 weeks. Secondary endpoints: Changes in CKD stage and safety profiles (platelets, lipids, AST, ALT, creatinine, eGFR) over time.

### Statistical analysis

Continuous variables were first assessed for normality and are presented as the mean ± standard deviation (SD) for normally distributed data or as the median with interquartile range (IQR) for non-normally distributed data. Between-group comparisons were performed using Student’s *t*-test or the Mann–Whitney U test, as appropriate. Categorical variables are expressed as numbers and percentages, and comparisons between groups were conducted using the chi-square test. All statistical analyses were performed using SPSS version 25.0 (IBM Corp., Armonk, NY, USA), and a two-sided *P*-value < 0.05 was considered statistically significant.

### Ethical approval

The study complied with the Declaration of Helsinki (1975) and was approved by the Ethics Committee of Yantai Qishan Hospital (Approval No. 202306). The Ethics Committee exempted written informed consent and approved the experimental protocol.

## Results

### Baseline characteristics

A total of 122 chronic hepatitis B (CHB) patients meeting inclusion criteria (normal ALT, age > 30 years, and HBV DNA positivity) were enrolled. The cohort had a mean age of 47.6 ± 9.8 years and a median HBV DNA level of 3.64 log10 IU/mL (IQR 2.93–5.35). HBV DNA levels were distributed as follows: ≤ 2,000 IU/mL (40.2%), 2,001–20,000 IU/mL (26.2%), and > 20,000 IU/mL (33.6%). Baseline renal function assessment revealed a mean eGFR of 106.4 ± 20.7 mL/min/1.73 m^2^, with CKD stage distribution as follows: stage 1 (81.1%), stage 2 (18.2%), and stage 3 (0.8%). Detailed baseline characteristics are summarized in Table 1. Stratification by HBeAg status (positive: *n* = 30; negative: *n* = 92) revealed statistically significant intergroup differences in baseline HBV DNA levels, eGFR, and creatinine (*P* < 0.05, Table 2).

**TABLE 1:** Baseline characteristics of patients before treatment.

Characterization	Total (*n* = 122)
Gender, count (male, %)	72 (59)
Age	47.61 ± 9.75
30–35	15 (12.3)
> 35	107 (87.7)
HbeAg ( > 1 S/CO)	
Negative	30 (24.6)
Positive	92 (75.4)
HBV DNA (log10IU/ml) (%)	3.64 (2.93, 5.34)
≤ 2000	49 (40.2)
2000–20000	32 (26.2)
> 20000	41 (33.6)
CKD Stages (%)	
1	99 (81.1)
2	22 (18.2)
3	1 (0.8)
ALT (U/L)	22.70 (16.85, 30.93)
AST (U/L)	22.00 (19.00, 27.00)
TBIL (umol/L)	15.20 (10.65, 18.19)
TC (mmol/L)	4.69 ± 0.92
TG (mmol/L)	0.96 (0.74, 1.43)
PLT (g/L)	192.03 ± 55.48
Cr (umol/L)	69.40 (58.95, 74.75)
eGFR ml/(min/1.73 m^∧^2)	105.525 (99.01, 113.40)

**TABLE 2:** Comparison of baseline characteristics of patients in HbeAg (+) and HbeAg (–) groups.

Characterization	HbeAg(+) (*n* = 30)	HbeAg(-) (*n* = 92)	*P*
HBV DNA (log10IU/ml)	6.24 (3.26, 7.08)	3.48 (2.86, 4.27)	**0.001**
ALT (U/L)	22.5 (18.48, 33.23)	22.70 (16.00, 30.25)	0.168
AST (U/L)	21.00 (18.03, 26.78)	22.55 (19.00, 27.00)	0.281
TC (mmol/L)	4.50 (4.09, 5.25)	4.64 (3.96, 5.30)	0.759
TG (mmol/L)	0.9 8 (0.72, 1.45)	0.95 (0.75, 1.43)	0.177
PLT (g/L)	200.40 ± 69.01	188.96 ± 49.77	0.41
Cr (umol/L)	63.55 (51.03, 70.53)	70.50 (62.40, 75.00)	**<0.001**
eGFR ml/ (min/1.73 m^∧^2)	107.31 (100.91, 120.47)	104.23 (94.27, 111.42)	**0.043**

*P*-values in bold denotes the value <0.05.

### Efficacy and safety at 48 weeks

Following 48 weeks of TAF therapy, 74.6% of patients (91/122) achieved HBV DNA conversion (*P* < 0.001). Minimal changes were observed in HBsAg/HBeAg seroconversion rates and CKD stage progression, though these differences lacked statistical significance. Safety parameters, including aminotransferases (ALT, AST) and lipid profiles, remained stable throughout the treatment period (Table 3).

**TABLE 3:** Overall patient efficacy and safety of TAF treatment for 48 weeks.

Items	Baseline	48w (*n* = 122)	*P*
HbsAg (>0.05IU/ml) (%)			> 0.999
Negative	122 (100)	121 (99.2)	
Positive	0	1 (0.8)	
HbeAg (>1S/CO) (%)			0.88
Negative	30 (24.6)	28 (23.0)	
Positive	92 (75.4)	94 (77.0)	
HBV DNA conversion (%)			**<0.001**
negative	0 (0)	31 (25.4)	
positive	122 (100)	91 (74.6)	
CKD Stages (%)			0.554
1	99 (81.1)	99 (81.1)	
2	22 (18.2)	21 (17.20)	
3	1 (0.8)	0	
4	0	1 (0.8)	
5	0	1 (0.8)	
ALT (U/L)	22.70 (16.85, 30.93)	21.00 (15.15, 27.1)	0.106
AST (U/L)	22.00 (19.00, 27.00)	21.00 (18.68, 24.85)	0.117
TC (mmol/L)	4.6 1 (3.98, 5.30)	4.59 (4.02, 5.18)	0.749
TG (mmol/L)	0.96 (0.74, 1.43)	1.01 (0.75, 1.33)	0.881
PLT (g/L)	192.03 ± 55.48	190.44 ± 53.33	0.828
eGFR ml/(min/1.73m^∧^2)	105.53 (99.01, 113.40)	100.42 (94.71, 112.35)	0.118

*P*-values in bold denotes the value <0.05.

Subgroup analysis demonstrated differential virologic responses: HBV DNA conversion rates were 63.3% (19/30) in HBeAg-positive patients versus 78.3% (72/92) in HBeAg-negative patients (*P* < 0.001). No significant intergroup differences were observed in other efficacy or safety endpoints (Table 4).

**TABLE 4:** Comparison of efficacy and safety of HbeAg grouped patients treated for 48 weeks.

	HbeAg(+)			HbeAg(–)			*P*
Items	Baseline	48w	*P*	Baseline	48w	*P*	
HbsAg (>0.05IU/ml) (%)			/			> 0.999	/
negative	30 (100)	30 (100)		92 (100)	91 (98.9)		
positive	0	0		0	1 (1.1)		
CKD Stages (%)			1.00			0.553	/
1	26 (86.7)	26 (86.7)		73 (79.3)	73 (79.3)		
2	4 (13.3)	4 (13.3)		18 (19.6)	17 (18.5)		
3	0	0		1 (1.1)	0		
4	0	0		0	1 (1.1)		
5	0	0		0	1 (1.1)		
ALT (U/L)	22.70 (18.23, 36.70)	20.50 (17.00, 28.28)	0.176	22.70 (16.00, 30.00)	21.60 (15.00, 27.10)	0.273	0.138
AST (U/L)	21.00 (18.08, 26.33)	20.30 (17.13, 25.68)	0.591	22.80 (19.08, 27.00)	21.00 (19.00, 24.35)	0.106	0.172
TC (mmol/L)	4.51 (4.11, 5.14)	4.28 (3.80, 5.41)	0.609	4.65 (3.95, 5.30)	4.62 (4.04, 5.18)	0.783	0.958
TG (mmol/L)	0.93 (0.68, 1.34)	0.89 (0.65, 1.21)	0.829	1.03 (0.76, 1.46)	1.05 (0.80, 1.40)	0.988	0.145
PLT (g/L)	206.00 (140.25, 260.00)	198.00 (163.00, 236.25)	0.850	189.00 (163.00, 222.25)	190.00 (153.50, 216.00)	0.766	0.72
eGFR ml/(min/1.73m^∧^2)	107.43 (101.05, 122.38)	102.10 (96.82, 116.35)	0.135	103.90 (94.76, 111.26)	100.42 (93.08, 111.81)	0.325	0.92

### Efficacy and safety at 96 weeks

A total of 80 participants completed the 96-week follow-up; there were no significant differences in baseline characteristics between the lost-to-follow-up group and this subgroup (Supplementary Table 1). Among 80 patients completing 96-week follow-up, HBV DNA conversion rates increased progressively from 48 to 96 weeks (*P* < 0.001). HBsAg and HBeAg seroconversion rates showed modest but non-significant improvements. Transaminase levels, lipid profiles, and renal safety markers (e.g., creatinine, eGFR) exhibited no clinically meaningful changes (Table 5).

**TABLE 5:** Efficacy and safety of overall patients treated with TAF for 96 weeks.

Items	Baseline	48w (*n* = 80)	96w (*n* = 80)	P
HbsAg (>0.05IU/ml) (%)				0.164
Negative	80 (100)	79 (98.8)	76 (96.2)	
Positive	0	1 (1.3)	3 (3.8)	
HbeAg (>1S/CO) (%)				0.704
negative	23 (28.8)	19 (23.8)	19 (23.8)	
positive	57 (71.3)	61 (76.3)	61 (76.3)	
HBV DNA conversion (%)				**<0.001**
negative	80 (100)	21 (26.3)	13 (16.3)	
positive	0	59 (73.8)	67 (83.8)	
CKD Stages (%)				0.694
1	62 (77.5)	61 (76.3)	64 (80)	
2	18 (22.5)	18 (22.5)	16 (20)	
5	0	1 (1.3)	0	
ALT (U/L)	22.90 (17.00, 31.00)	22.00 (16.00, 30.50)	20.0 0 (15.85, 28.85)	0.393
AST (U/L)	21.5 5 (19.00, 2700)	21.00 (18.00, 25.48)	21.90 (18.00, 26.58)	0.722
TC (mmol/L)	4.80 (4.14, 5.36)	4.67 (4.14, 5.42)	4.62 (4.02, 5.12)	0.714
TG (mmol/L)	0.98 (0.77, 1.40)	1.02 (0.77, 1.33)	1.00 (0.83, 1.42)	0.857
PLT (g/L)	190.03 ± 59.23	186.39 ± 55.96	187.26 ± 54.13	0.917
eGFR ml/(min/1.73 m^∧^2)	104.17 (93.85, 112.89)	104.25 (94.94, 116.85)	103.86 (87.57, 115.38)	0.897

*P*-values in bold denotes the value <0.05.

In HBeAg-stratified analysis, HBV DNA conversion rates escalated significantly over time (*P* < 0.001), reaching 56.5% (13/23) in HBeAg-positive patients and 94.7% (54/57) in HBeAg-negative patients at 96 weeks. Intergroup comparisons of secondary endpoints revealed no statistically significant differences (Table 6).

**TABLE 6:** Comparison of efficacy and safety of 96 weeks of treatment in HbeAg grouped patients.

	HbeAg(+)				HbeAg(–)				*P*
Items	Baseline	48w	96w	*P*	Baseline	48w	96w	*P*	
HbsAg (>0.05IU/ml) (%)				/				0.167	/
Negative	23 (100)	23 (100)	22 (100)		57 (100)	56 (98.2)	54 (94.7)		
Positive	0	0	0		0	1 (1.8)	3 (5.3)		
CKD Stages (%)				0.910				0.616	/
1	19 (82.6)	19 (82.6)	18 (78.3)		43 (75.4)	42 (73.7)	46 (80.7)		
2	4 (17.4)	4 (17.4)	5 (21.7)		14 (24.6)	14 (24.6)	11 (19.3)		
5	0	0	0		0	1 (1.8)	0		
ALT (U/L)	22.00 (17.00, 30.90)	21.70 (16.90, 33.20)	20.00 (12.00, 28.40)	0.239	23.00 (16.50, 31.50)	22.00 (15.60,2 9.55)	20.00 (16.05, 30.50)	0.775	0.848
AST (U/L)	20.10 (18.00, 26.10)	18.90 (17.00,25.60)	22.00 (15.00, 26.20)	0.807	22.70 (19.15, 27.10)	21.00 (19.00, 26.05)	21.80 (18.00, 26.85)	0.753	0.269
TC (mmol/L)	4.859 (4.13, 5.36)	4.44 (4.04, 5.67)	4.60 (4.02, 5.58)	0.997	4.85 (4.09, 5.38)	4.68 (4.17, 5.41)	4.62 (4.03, 5.09)	0.501	0.471
TG (mmol/L)	0.94 (0.77, 1.40)	0.98 (0.75, 1.29)	0.97 (0.86, 1.66)	0.685	1.08 (0.78, 1.41)	1.05 (0.78, 1.33)	1.00 (0.83, 1.42)	0.990	0.833
PLT (g/L)	195.26 ± 72.37	195.26 ± 65.85	190.05 ± 66.34	0.959	187.84 ± 53.40	182.46 ± 51.21	186.06 ± 48.72	0.859	0.939
eGFR ml/(min/1.73 m^∧^2)	101.81 (94.09, 110.14)	101.28 (92.42,107.66)	103.53 (96.50, 121.80)	0.667	105.10 (93.04, 113.93)	105.76 (95.87, 118.75)	104.20 (85.12, 115.05)	0.611	0.067

## Discussion

Hepatitis B virus infection is a global public health problem, and various countries and regions are gradually clarifying the direction and scope of its treatment in order to minimize the disease progression and adverse events caused by the virus infection, so that more patients can benefit from it. In previous guidelines, except for some special cases, patients with normal aminotransferases were not considered for antiviral therapy. However, in actual studies (13), it has been found that about 1/3 or more of patients with normal ALT have significant histologic changes in the liver, suggesting that there is a risk of disease progression and that antiviral therapy should be given. A substantial proportion of patients with chronic hepatitis B (CHB) fall into the “indeterminate phase” that lies outside conventional natural history classifications, yet no universally established guidelines exist for initiating antiviral therapy in this population. Notably, multiple clinical studies focusing on HBeAg-positive patients have demonstrated significant clinical benefits from antiviral intervention (14, 15). Supporting this perspective, a nationwide Korean cohort study (16) revealed that expanding antiviral treatment eligibility substantially reduced HBV-related morbidity and mortality burdens. Although our study did not track long-term survival outcomes, it demonstrated comparable efficacy in achieving virological clearance rates. Furthermore, our investigation extended the comparative analysis to the understudied HBeAg-negative population, a demographic frequently excluded from prior therapeutic evaluations.

In China’s updated clinical guidelines for managing chronic hepatitis B patients with positive serum HBV DNA and normal alanine aminotransferase (ALT), the inclusion of age > 30 years as an independent treatment criterion represents a significant advancement from the 2019 version, where age consideration was contingent upon hepatic fibrosis status. Our pioneering 96-week longitudinal study systematically evaluates the therapeutic efficacy and safety profiles in this newly defined patient cohort, providing real-world evidence for age-based treatment models and supporting the use of age as an independent determinant in decisions regarding antiviral therapy.

In actual clinical practice, most patients with hepatitis B have no obvious clinical symptoms and are reluctant to undergo liver puncture biopsy to clarify pathological changes, which requires us to use more noninvasive means to assess whether patients have inflammatory changes or fibrotic lesions in the liver to determine the timing of antiviral therapy. Age as an initial assessment indicator is instructive in the selection of antiviral therapy. A higher percentage of patients over 40 years of age were found to have inflammatory liver changes (5, 17). The AASLD guidelines also state that patients aged > 40 years are more likely to have significant histologic liver disease and antiviral therapy is recommended. An age-time cohort analysis of trends in liver disease deaths over 30 years in mainland China found (10) that the risk of liver disease deaths increased dramatically after patients were older than 30 years of age, all of which reflects the fact that age is a key factor influencing disease change. Based on this, the latest guideline for hepatitis B in China has added patients who fulfill the criteria of serum HBV DNA positivity, normal ALT, and age > 30 years to the population eligible for antiviral therapy, and our study will further confirm the effectiveness of antiviral therapy in this population.

The clearance rate of HBV DNA serves as a pivotal indicator for evaluating the therapeutic efficacy of oral antiviral agents. Previous investigations into antiviral treatment outcomes and prognosis in chronic hepatitis B (CHB) patients have predominantly conducted separate analyses based on HBeAg status. These studies consistently demonstrated favorable treatment responses and reduced complication rates across study populations following antiviral therapy (18). Our findings similarly revealed a progressive annual increase in HBV DNA clearance rates under TAF therapy, observed both in the overall cohort and HBeAg-stratified subgroups. Notably, HBeAg-negative patients achieved the highest clearance rate of 94.7% at week 96. Although statistical comparison between HBeAg-positive and negative groups was precluded by baseline characteristic disparities, the final data suggested comparable HBV DNA clearance rates at both 48 and 96 weeks of TAF treatment, with a potential trend toward superior efficacy in HBeAg-negative patients—a hypothesis requiring further large-scale validation. Gao et al. also support the use of TAF for antiviral treatment in patients with normal ALT levels. The patients included in their studies showed better results when their viral load was low ( < 2000 IU/mL), and the 12-week virological response rate reached 100% (11), indicating that expanding the indications for antiviral treatment may help control the global burden of hepatitis B. Qian Rongnan et al. (19) propose that persistent HBV replication constitutes the primary driver of hepatic necroinflammation and disease progression. This pathogenic mechanism underscores the fundamental therapeutic objective in CHB management: sustained viral suppression to prevent hepatic fibrosis, cirrhosis, hepatocellular carcinoma, and ultimately improve long-term survival. Our findings reinforce the clinical imperative for continuous antiviral therapy regardless of HBeAg status, as achieving HBV DNA seroclearance remains crucial for optimizing hepatic outcomes.

In addition, TAF significantly inhibits HBV DNA but has no direct effect on cccDNA in HBV-infected hepatocytes, and continued long-term therapy is usually required to maintain HBV suppression, but the ultimate goal of HBsAg clearance is rarely achieved. Therefore, it has been suggested (20) that antiviral drug therapy should be appropriately discontinued in HBeAg-negative patients to save costs. However, in our subgroup comparison, we found that HBsAg clearance was still achieved in a very small number of patients under TAF treatment, and all of them occurred in HBeAg-negative patients, up to 5.3% at 96 weeks of treatment, which, although the results were not statistically significant, contributed greatly to the data on hepatitis B cure, and should be positively recommended to the treatment of HBeAg-negative patients (21). Therefore, we advocate the effectiveness of TAF treatment and the worthiness of long-term adherence to follow-up.

The safety of tenofovir alafenamide (TAF) remains a critical consideration. Under the AASLD criteria for ALT normalization (male: ALT ≤ 30 U/L; female: ALT ≤ 19 U/L), TAF demonstrated a superior ALT normalization rate compared to tenofovir disoproxil fumarate (TDF). Notably, all patients in our cohort maintained normal ALT levels throughout the treatment period, further validating the safety and efficacy of TAF. In addition, there was a greater safety profile for the kidneys and bone mineral density in patients using TAF (22, 23). Regarding renal and bone safety parameters, TAF exhibited a favorable profile. Longitudinal monitoring of estimated glomerular filtration rate (eGFR) and chronic kidney disease (CKD) staging over 96 weeks revealed no statistically significant changes in the overall cohort (*p* > 0.05). Subgroup analyses stratified by HBeAg status (positive vs. negative) yielded consistent findings, with no clinically relevant alterations in renal parameters observed. These data collectively underscore the renal safety of TAF across different HBeAg statuses. While potential concerns exist about TAF’s impact on lipid metabolism (24), our serial assessments of triglyceride and total cholesterol levels demonstrated no significant fluctuations during treatment (*p* > 0.05). This observation suggests that TAF does not adversely affect lipid profiles in our study population, reinforcing its overall safety advantage.

Of course, our study still has some limitations. First, retrospective studies may have some confounding factors, such as selection bias, center effects, and lack of comparator arm, affecting the experimental results. Second, the therapeutic effect of TAF on this population was studied only in terms of virologic response, however, due to the limited follow-up period, the cases of HBsAg and HBeAg turning negative were not observed, and the changes in the degree of liver fibrosis during the treatment of patients were not addressed. Furthermore, this study also lacks the genotype data of the patients, so the relationship between antiviral efficacy and genotype has not yet been clarified. In addition, renal function is currently assessed using only two indicators—creatinine and eGFR—and more renal function-related indicators will need to be incorporated in the future to comprehensively evaluate the renal safety of TAF. We hope to strengthen the comparison of data on the clinical prognosis of patients’ livers in future studies and to extend the follow-up period as much as possible to explore the treatment effects on patients in the longer term.

## Conclusion

In the hepatitis B population with normal ALT, positive HBV DNA and age > 30 years, including HBeAg-positive and negative patients, adherence to long-term TAF treatment has obvious efficacy, which can significantly inhibit viral replication and has better renal safety. Although TAF showed promising virological suppression and acceptable short- to mid-term renal safety in this cohort, larger comparative studies are still needed.

## Data Availability

The original contributions presented in this study are included in this article/Supplementary material, further inquiries can be directed to the corresponding author.
