# Supplementary material for: Efficacy and safety of tenofovir alafenamide in HBeAg-positive and negative chronic hepatitis B patients aged over 30 years with normal ALT and detectable HBV DNA: a 96-week retrospective two-center study
Source: Front Med (Lausanne). 2026 Jul 23;13:1764956. doi: 10.3389/fmed.2026.1764956 (PMC13441701; doi:10.3389/fmed.2026.1764956)
Supplement: Supplementary file 1 [file Table_1.docx]

Table S1

| Characterization | Follow up to 96W(n=80) | Loss to follow up (n=42) | Statistical magnitude | P |
| --- | --- | --- | --- | --- |
| Gender, count (male, %) | 47(41.3) | 25（40.5） | F=0.007 | 0.935 |
| Age | 48.34±9.86 | 46.21±9.50 | T=-1.145 | 0.255 |
| 30-35 | 6(7.5) | 9(21.4) |  |  |
| >35 | 74(92.5) | 33(78.6) |  |  |
| HbeAg (>1 S/CO) |  |  | F=2.172 | 0.143 |
| negative | 57(71.3) | 32(83.3) |  |  |
| positive | 23(28.8) | 7(16.7) |  |  |
| HBV DNA (log10IU/ml) (%) | 3.53(2.77,5.00) | 3.86(3.19,5.63) | Z=-1.544 | 0.123 |
| ≤2000 | 35(43.8) | 14(33.3) | F=1.634 | 0.204 |
| 2000-20000 | 21(26.3) | 11(26.2) |  |  |
| >20000 | 24(30.0) | 17(40.5) |  |  |
| CKD Stages (%) |  |  | F=1.057 | 0.306 |
| 1 | 62(77.5) | 37(88.1) |  |  |
| 2 | 18(22.5) | 4(9.5) |  |  |
| 3 | 0 | 1(2.4) |  |  |
| ALT (U/L) | 24.42±8.72 | 23.31±8.15 | Z=-0.434 | 0.664 |
| AST (U/L) | 23.80±8.14 | 22.91±4.70 | Z=-0.248 | 0.804 |
| TC (mmol/L) | 4.79±0.97 | 4.46±0.78 | t=-1.615 | 0.110 |
| TG (mmol/L) | 1.24±0.97 | 1.21±0.82 | Z=-0.489 | 0.625 |
| PLT (g/L) | 190.03±59.23 | 196.62±46.22 | t=0.576 | 0.565 |
| Cr (umol/L) | 67.45±12.09 | 71.91±24.44 | Z=-.0795 | 0.427 |
| eGFR ml/(min/1.73m^2) | 108.31±23.09 | 102.70±14.70 | Z=-0.509 | 0.611 |
